# Impact of atherosclerotic cardiovascular disease on healthcare resource utilization and costs in patients with type 2 diabetes mellitus in a real-world setting

**DOI:** 10.1186/s40842-019-0090-y

**Published:** 2020-03-04

**Authors:** Wayne Weng, Ye Tian, Sheldon X. Kong, Rahul Ganguly, Malene Hersloev, Jason Brett, Todd Hobbs

**Affiliations:** Novo Nordisk Inc., 800 Scudders Mill Road, Plainsboro, NJ 08536 USA

**Keywords:** Atherosclerotic cardiovascular disease, Type 2 diabetes mellitus, Healthcare utilization, Cost, Real-world, Claims

## Abstract

**Background:**

This study evaluated the impact of atherosclerotic cardiovascular disease (ASCVD) on healthcare resource utilization and costs in patients with type 2 diabetes mellitus (T2DM).

**Methods:**

This study was a retrospective, cross-sectional study using US claims data. Adult patients with T2DM were stratified by presence or absence of ASCVD and compared regarding annual (2015) healthcare resource utilization and associated costs. Subgroup analyses were conducted for three age groups (18–44, 45–64, and ≥ 65 years).

**Results:**

Among 1,202,596 eligible patients with T2DM, 45.2% had documented ASCVD. The proportions of patients with inpatient and ER-based resource utilization during 2015 were three-to-four times greater in the ASCVD cohort as compared to the non-ASCVD cohort for the categories of inpatient visits (15.6% vs 4.4% of patients), outpatient ER visits (18.4% vs 5.2% of patients), and inpatient ER visits (4.3% vs 0.9% of patients). Outpatient utilization also was higher among patients with ASCVD as compared to those without ASCVD (mean number of annual office visits per patient, 9.1 vs 5.6), and more than twice as many patients with ASCVD had ≥ 9 office visits (43.5% vs 19.8%). Average per-patient total healthcare cost was $22,977 for ASCVD vs $9735 for non-ASCVD, with medical costs primarily driving the difference ($17,849 vs $6079); the difference in pharmacy costs was smaller ($5128 vs $3656). In the 18–44, 45–64, and ≥ 65 age subgroups respectively, total annual healthcare costs were 143, 127, and 114% higher in ASCVD vs non-ASCVD patients.

**Conclusions:**

These findings indicate significantly higher healthcare resource utilization and associated costs in patients having T2DM with ASCVD compared to T2DM without ASCVD.

## Background

Diabetes mellitus is a highly prevalent disease associated with a large economic burden for patients and healthcare systems. The number of people estimated to be affected by diabetes worldwide in 2017 was 451 million and this number is expected to increase to 693 million by 2045 [1]. In the United States, the prevalence of diabetes in 2015 was estimated to be 30.2 million adults, or 12.2% of all US adults. The prevalence of diabetes increases with age and was estimated to affect 25.2% of individuals aged 65 and over in the US in 2015 [2]. If current trends continue, 33% of adults in the US could have diabetes by 2050 [3]. Type 2 diabetes mellitus (T2DM) accounts for 95% of diabetes cases in the US [2]. Medical costs associated with treating US patients with diabetes reflect this high prevalence; approximately 1 out of every 4 healthcare dollars in the US in 2012 ($306 billion) were attributed to people with diabetes [4].

Diabetes is associated with multiple comorbidities including cardiovascular disease (CVD), which is one of the most prevalent causes of T2DM-related morbidity and mortality in both men and women [5, 6]. Comorbidities in general, and CVD specifically, can add significantly to the economic burden of diabetes. Understanding the economic burden of CVD in patients with T2DM is important to inform the development of holistic and cost-effective prevention and management strategies for T2DM. A recent systematic review of global data reported that the presence of CVD increases annual costs in patients with T2DM by $3418 to $9705 (2016 US$) as compared to patients having T2DM without CVD [7]. Nationwide data on the impact of CVD on overall treatment costs in patients with T2DM in the US are somewhat limited. Prior studies have been regional [8, 9], not focused on total healthcare costs [10, 11], or included relatively small datasets for studies of this nature [12]. A recent study by Mehta et al., utilizing a national linked electronic medical records (EMR) and claims database, evaluated data on 138,018 adults with T2DM and found significantly higher total medical costs in those with a history of CVD versus those without [13].

Beginning in 2016, the American Diabetes Association treatment guidelines have focused specifically on “atherosclerotic cardiovascular disease” (ASCVD), defined as acute coronary syndromes, a history of myocardial infarction (MI), stable or unstable angina, coronary or other arterial revascularization, stroke, transient ischemic attack, or peripheral arterial disease presumed to be of atherosclerotic origin [14]. The current study was designed to further the understanding of the impact of ASCVD on healthcare resource utilization and costs in patients with T2DM in the United States (US) using a large (1 million+) population identified within a national claims database. The clinical characteristics and antidiabetes treatment patterns of this population have been analyzed separately and reported elsewhere [15].

## Methods

### Study cohorts

This study was designed as a retrospective, cross-sectional analysis of a large US administrative claims database (IBM® Family of MarketScan® Research Databases, formerly Truven Health Analytics MarketScan Databases) which has captured data from employer-sponsored health insurance plans since 1995 [16]. The MarketScan database contains individual healthcare claims data of more than 245 million unique patients from all 50 states in the US. All data were de-identified and fully compliant with the Health Insurance Portability and Accountability Act of 1996.

Data were included in the analysis for all patients in the database with an established T2DM diagnosis before January 1, 2015 as determined by (1) at least two diagnoses for T2DM based on international classification of diseases, ninth revision (ICD-9) codes of 250.×0 or 250.×2 or ICD-10 codes of E11.xx, or at least one T2DM diagnosis and at least one oral antidiabetic drug claim and (2) no more than one type 1 diabetes mellitus diagnosis (allowing for the possibility of one mis-coding) based on ICD-9 (250.×1, 250.×3) or ICD-10 (E10,x) codes. Additionally, patients were required to be at least 18 years old on January 1, 2015 with continuous health plan enrollment with an insurance plan containing both medical and pharmacy benefits between January 1, 2014 and December 31, 2015. Eligible patients were stratified into two groups based upon the presence or absence of ASCVD before December 31, 2015: without ASCVD (non-ASCVD) and with ASCVD (ASCVD). ASCVD was defined based on relevant ICD-9/-10 codes corresponding to diagnoses included in the ADA 2017 definition of ASCVD, including: acute coronary syndrome, history of MI, stable or unstable angina, peripheral arterial disease presumed to be of atherosclerotic origin, stroke, transient ischemic attack, and coronary or other arterial revascularization [17]. A detailed listing of relevant ICD-9/-10 codes has been provided elsewhere [15].

### Variables

Patient demographics (age and sex) and baseline data were recorded as of January 1, 2015. Baseline data included geographic region (North Central, Northeast, South, West, or unknown), and insurance (commercial or Medicare). Comorbidities were captured using ICD-9 and ICD-10 codes in claims data for the years 2014 and 2015 (any appearance). Diabetes Complications Severity Index (DCSI) scores [18] and Charlson Comorbidity Index (CCI) scores [19] were determined based on the comorbidities identified for each patient. The CCI, designed to be a prediction of 10-year survival, factors in patient age plus the presence/absence of the following comorbidities: MI, congestive heart failure, peripheral vascular disease, cerebrovascular accident/transient ischemic attack, dementia, chronic obstructive pulmonary disease, connective tissue disease, peptic ulcer disease, liver disease, diabetes mellitus, hemiplegia, moderate to severe chronic kidney disease, solid tumor, leukemia, lymphoma, and AIDS. The DCSI is a score based on the presence of specific comorbidities included in the following categories: retinopathy, nephropathy, neuropathy, cerebrovascular, cardiovascular, peripheral vascular disease, and metabolic (see Additional file 1: Table S1 for listing and scoring values of specific diagnoses). The comorbidities of hypertension and dyslipidemia were also evaluated, as these were not part of the ASCVD definition, nor were they reflected in the DCSI or CCI determinations.

Healthcare resources used by eligible patients in 2015 were evaluated according to the number and types of healthcare visits in the following categories: all outpatient office visits**,** outpatient visits to endocrinology and cardiology specialists, inpatient hospital admissions, and emergency room (ER) visits that did (inpatient ER) and did not (outpatient ER) result in hospital admission. Healthcare costs incurred by eligible patients in 2015 were determined in total for individual medical (outpatient, inpatient, and ER visits) and pharmacy cost components. Some patients in the MarketScan database had health insurance, which is not fee-for-service (health insurance provider pays a fixed annual fee per patient); these patients were not eligible for the cost analyses. Cost data in the MarketScan database are based on fully paid and adjudicated claims [16]. Costs reflect payments made by health insurance companies and included individual items for outpatient claims (eg, office visits and associated laboratory tests, medical procedures performed in a doctor’s office, outpatient prescription drugs) or as an aggregated payment (all services provided) for ER visits and inpatient hospital stays.

### Analyses

Continuous data were summarized by mean, standard deviation (SD), and count; categorical data by number and percentage. Subgroup analyses by age group (18–44, 45–64, ≥ 65 years) were performed for the prevalence of ASCVD among patients with T2DM and for average annual (2015) healthcare costs per patient (medical, pharmacy, and total costs) by ASCVD status.

A sensitivity analysis was performed on 1:1 propensity score matched ASCVD vs non-ASCVD cohorts. The propensity score matching was based on a logistic regression model adjusted for age, sex, region, and insurance type. We then used a linear regression model adjusted for age, sex, region, and insurance type to estimate the total healthcare costs for the matched ASCVD and non-ASCVD cohorts. Propensity score matching was done using the SAS PSMATCH procedure with the greedy nearest neighbor match without replacement and a caliper width of 0.2 of the SD of the logit of the propensity score as proposed by Austin [20].

## Results

### Study population

**Fig. 1:**
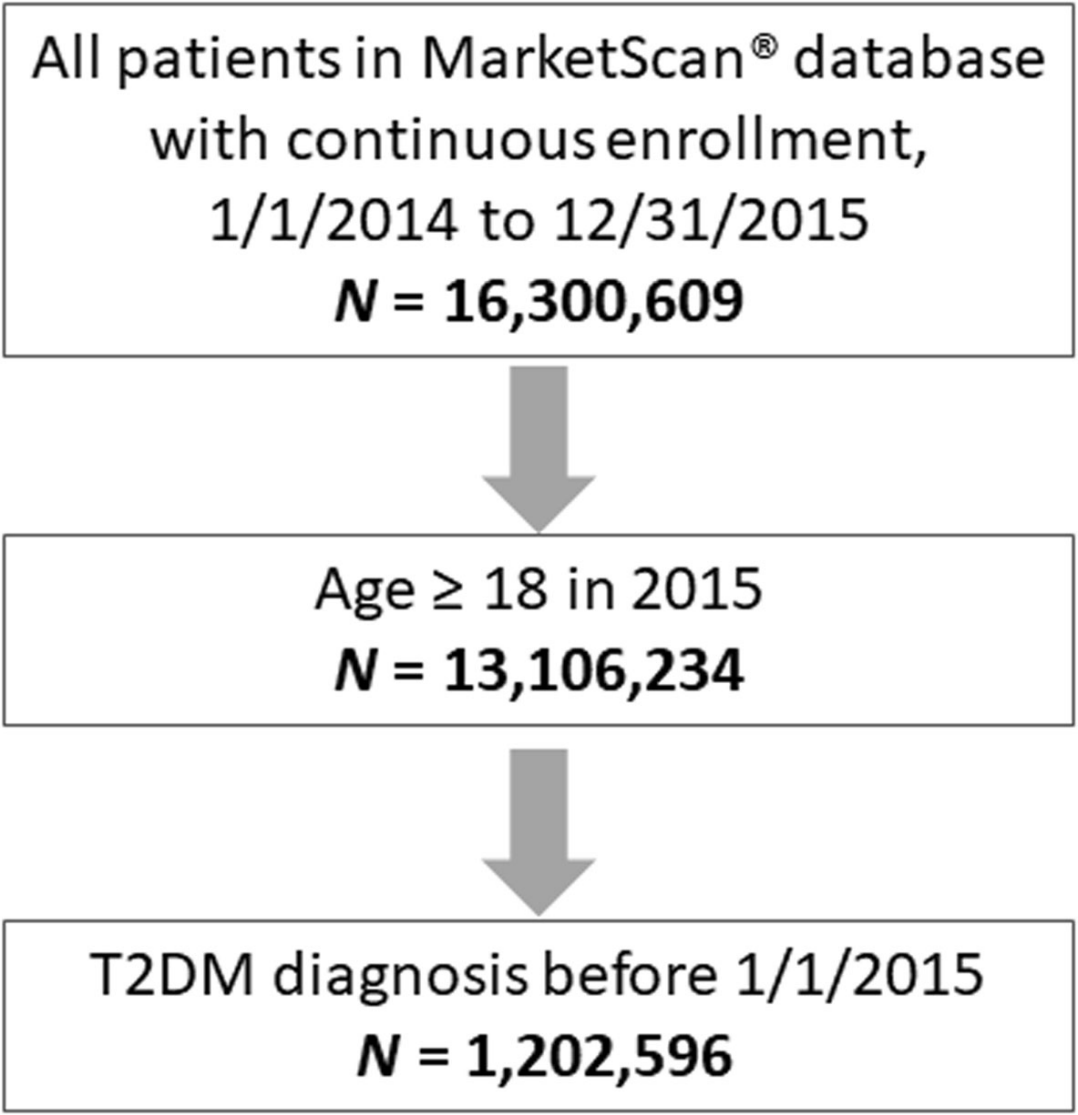
Derivation of study population. *T2DM* Type 2 diabetes mellitus

**Fig. 2:**
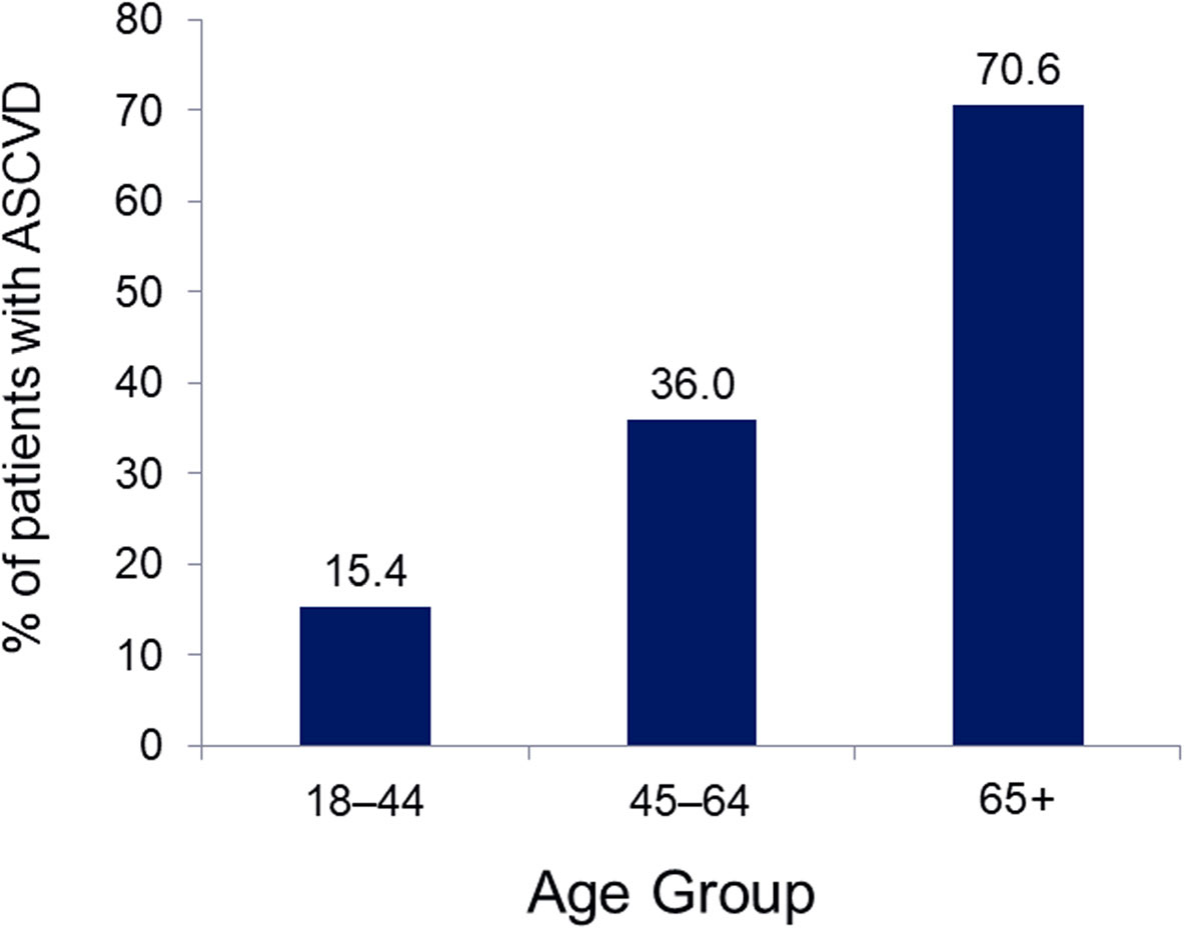
ASCVD^a^ prevalence in a real-world T2DM population in the US in 2015 by age group. Footnotes: *ASCVD* Atherosclerotic cardiovascular disease, *T2DM* Type 2 diabetes mellitus. ^a^As defined by ADA 2017 guidelines

There were 16,300,609 patients within the MarketScan database having continuous enrollment from January 1, 2014 through December 31, 2015; of these, 1,202,596 met the study inclusion criteria (Fig. 1) and were included in the analysis population (Table 1). The mean age of patients eligible for analysis was 60.9 years. Slightly less than half (45.2%) of eligible patients had documented ASCVD. The mean age of patients with ASCVD (66.5 years) was higher than patients without ASCVD (56.2 years). There were no meaningful differences between groups with regard to sex and geographic distribution (Table 1), although a higher percentage of patients with ASCVD lived in the North Central US and fewer in the South and West as compared to the cohort of patients without ASCVD. The proportion of patients with ASCVD increased with increasing age category, from 15% among those aged 18–44 years to 36% among those aged between 45 and 64 years to 71% among those older than 65 years of age (Fig. 2).

**Table 1:** Demographics and clinical characteristics of a real-world type 2 diabetes mellitus population in the US in 2015, stratified by ASCVD status

Patients with type 2 diabetes mellitus			
Variable	Non-ASCVD *N* = 659,498 (54.8%)	ASCVD *N* = 543,098 (45.2%)	All patients *N* = 1,202,596 (100.0%)
Age, y, mean (SD)	56.2 (11.3)	66.5 (12.3)	60.9 (12.8)
Age category, n (%)			
18–44 y	93,646 (14.2)	17,030 (3.1)	110,676 (9.2)
45–64 y	452,819 (68.7)	254,453 (46.9)	707,272 (58.8)
≥ 65 y	113,033 (17.1)	271,615 (50.0)	384,648 (32.0)
Sex, n (%)			
Female	335,295 (50.8)	255,579 (47.1)	590,874 (49.1)
Male	324,203 (49.2)	287,519 (52.9)	611,722 (50.9)
Region of US, n (%)			
North Central	143,870 (21.8)	172,345 (31.7)	316,215 (26.3)
Northeast	123,048 (18.7)	112,886 (20.8)	235,934 (19.6)
South	297,423 (45.1)	208,094 (38.3)	505,517 (42.0)
West	93,867 (14.2)	48,701 (9.0)	142,568 (11.9)
Unknown	1290 (0.2)	1072 (0.2)	2362 (0.2)
Insurance, n (%)			
Commercial	553,676 (84.0)	274,389 (50.5)	828,065 (68.9)
Medicare	105,822 (16.1)	268,709 (49.5)	374,531 (31.1)
ASCVD diagnosis^a^, n (%)			
Acute coronary syndrome	–	319,931 (58.9)	319,931 (26.6)
Angina	–	111,209 (20.5)	111,209 (9.3)
MI	–	89,498 (16.5)	89,498 (7.4)
Peripheral arterial disease	–	294,092 (54.2)	294,092 (24.5)
Revascularization	–	93,365 (17.2)	93,365 (7.8)
Stroke	–	223,736 (41.2)	223,736 (18.6)
Transient ischemic attack	–	76,790 (14.1)	76,790 (6.4)
Hypertension, n (%)	472,299 (71.6)	478,642 (88.1)	950,941 (79.1)
Dyslipidemia, n (%)	484,175 (73.4)	450,792 (83.0)	934,967 (77.8)
Diabetes-related complications^b^, n (%)			
Cardiovascular^c^	23,545 (3.6)	311,388 (57.3)	334,933 (27.9)
Cerebrovascular	0 (0.0)	118,557 (21.8)	118,557 (9.9)
Metabolic^d^	79,359 (12.0)	69,721 (12.8)	149,080 (12.4)
Nephropathy	61,975 (9.4)	121,068 (22.3)	183,043 (15.2)
Peripheral vascular^e^	14,388 (2.2)	111,131 (20.5)	125,519 (10.4)
Retinopathy	63,101 (9.6)	82,427 (15.2)	145,528 (12.1)
DCSI score, mean (SD)	0.77 (1.2)	2.7 (2.3)	1.65 (2.0)
CCI score, mean (SD)	1.7 (1.4)	3.3 (2.4)	2.4 (2.1)

Note: Age, sex, region, and insurance determined as of January 1, 2015. Comorbidities were captured by any appearance during 2014–2015

*ASCVD* Atherosclerotic cardiovascular disease, *CCI* Charlson Comorbidity Index, *DCSI* Diabetes Complications Severity Index, *MI* Myocardial infarction, *SD* standard deviation

^a^Defined by ADA 2017 guidelines. Patients could have more than one diagnosis

^b^Comorbidities included in the Diabetes Complications Severity Index [18]

^c^As defined by the Diabetes Complications Severity Index [18], category includes diagnoses of atherosclerosis, other ischemic heart disease, angina, other chronic ischemic heart disease, MI, ventricular fibrillation, arrest; atrial fibrillation, arrest; other ASCVD, old MI, heart failure, atherosclerosis, severe; aortic aneurysm/dissection

^d^Category includes ketoacidosis, hyperosmolar, and “other coma”

^e^Category includes any peripheral vascular disease, not limited to “peripheral arterial disease presumed to be of atherosclerotic origin” which was part of the “ASCVD” definition

The proportion of patients with Medicare was higher among patients with ASCVD than among those without ASCVD (49.6% vs 16.1%) and closely mirrored the proportion of patients older than 64 years in each group (50.5% vs 17.1%). Among patients with ASCVD, the most common ASCVD diagnoses were acute coronary syndrome (58.9%), peripheral arterial disease (54.2%), and stroke (41.2%); all other ASCVD diagnoses were noted in 14–20% of patients (Table 1). Hypertension and dyslipidemia were highly prevalent in both cohorts, but more prevalent among patients with ASCVD (88.1 and 83.0%, respectively) than among patients without ASCVD (71.6 and 73.4%, respectively). All diabetes-related complications were more prevalent among patients with ASCVD as compared to those without ASCVD (Table 1). The most common diabetes-associated complication was cardiovascular, noted in 57.3% of those with ASCVD (per study inclusion definitions) and 3.6% of those without ASCVD. The most common diabetes-associated complication in patients without ASCVD was metabolic (12.0%), which was the least prevalent diabetes-associated complication in patients with ASCVD (12.8%). As measured by DCSI and CCI scores, patients with ASCVD had greater disease complication and comorbidity burden as compared to those without ASCVD. Mean (SD) DCSI scores for patients with and without ASCVD were 2.7 (2.3) and 0.8 (1.2), respectively; corresponding CCI scores were 3.3 (2.4) and 1.7 (1.4) (Table 1).

### Healthcare resource utilization

Overall, 94% of patients had at least one outpatient office visit during 2015, with little difference noted between those with ASCVD (95.6%) and those without ASCVD (93.2%). However, patients with ASCVD used more physician resources than did patients without ASCVD. On average, patients with ASCVD had 63% more outpatient office visits as compared to those without ASCVD (mean number of annual visits per patient, 9.1 vs 5.6); 71.6% of patients with ASCVD had 5 or more office visits compared to 49.6% in patients without ASCVD, and more than twice as many patients with ASCVD had 9 or more office visits than did patients without ASCVD (43.5% vs 19.8%) (Table 2). As expected, a larger percentage of patients with ASCVD were seen by cardiology specialists as compared to patients without ASCVD (40.0% vs 11.1%), but endocrinology specialist visits were low in both the ASCVD and non-ASCVD groups (8.7% vs 8.0%).

**Table 2:** Healthcare resource utilization in a real-world type 2 diabetes mellitus population in the US in 2015, stratified by ASCVD status

Visit	Patients with type 2 diabetes mellitus	
	Non-ASCVD *N* = 659,498 (54.8%)	ASCVD *N* = 543,098 (45.2%)
Outpatient office visit		
Visits/patient, mean (SD)	5.6 (5.0)	9.1 (7.3)
Patients with visits, n (%)	614,582 (93.2)	518,969 (95.6)
1 to 4 visits	293,706 (44.5)	129,764 (23.9)
5 to 8 visits	190,109 (28.8)	152,856 (28.2)
9+ visits	130,767 (19.8)	236,349 (43.5)
Inpatient hospital visit		
Visits/patient, mean (SD)	0.1 (0.3)	0.2 (0.6)
Patients with visits, n (%)	29,194 (4.4)	84,905 (15.6)
1 visit	25,428 (3.9)	66,343 (12.2)
2+ visits	3766 (0.6)	18,562 (3.4)
Emergency room, outpatient^a^		
Visits/patient, mean (SD)	0.2 (0.8)	0.6 (1.5)
Patients with visits, n (%)	100,442 (15.2)	154,220 (28.4)
1 visit	69,787 (10.6)	85,999 (15.8)
2+ visits	30,655 (4.7)	68,221 (12.6)
Emergency room, inpatient^b^		
Visits/patient, mean (SD)	0.0 (0.1)	0.1 (0.2)
Patients with visits, n (%)	5627 (0.9)	23,180 (4.3)
1 visit	5274 (0.8)	20,898 (3.9)
2+ visits	353 (0.1)	2282 (0.4)
Visits by specialty, n (%)		
Endocrinology	52,745 (8.0)	47,011 (8.7)
Cardiology	73,286 (11.1)	217,159 (40.0)

*ASCVD* Atherosclerotic cardiovascular disease

^a^Not resulting in inpatient admission

^b^Resulting in inpatient admission

Patients with ASCVD also used more hospital and ER resources than patients without ASCVD (Table 2). In the ASCVD cohort, 15.6% of patients had an inpatient hospital visit during 2015, which was about 3.5 times greater than the percentage of patients in the non-ASCVD group who had an inpatient visit (4.4%). The percentage of patients who had multiple inpatient hospital visits during 2015 was 6 times greater in the with-ASCVD vs non-ASCVD cohort (3.4% vs 0.6%) (Table 2). Patients with ASCVD were almost twice as likely as patients without ASCVD to have an outpatient ER visit (28.4% vs 15.2%). The proportion of patients experiencing an inpatient ER visit was also greater in the ASCVD cohort than it was in the non-ASCVD cohort (4.3% vs 0.9%).

### Healthcare costs

Healthcare cost data were available from 474,271 (87.3%) patients with ASCVD and from 562,185 (85.2%) patients without ASCVD (Table 3). For all patients with cost data, the mean annual total healthcare cost per each patient with T2DM in 2015 was $15,794. For patients with ASCVD, mean annual total healthcare costs per patient were 2.4-fold higher than for patients without ASCVD ($22,977 vs $9735); the greatest category driver was medical costs, which were 2.9-fold higher for patients with ASCVD than for patients without ASCVD ($17,849 vs $6079) (Table 3). Mean annual pharmacy costs per patient were $1472 higher for patients with ASCVD as compared to patients without ASCVD ($5128 vs $3656). Mean annual outpatient office visit and inpatient hospital visit costs in 2015 were $6072 ($10,379 vs $4227) and $4334 ($5646 vs $1312) higher, respectively, for patients with ASCVD than for patients without ASCVD. ER-related costs contributed the least to total annual costs, but were also higher in the ASCVD vs the non-ASCVD cohort for both ER outpatient visits ($925 vs $382) and ER inpatient visits ($928 vs $158) (Table 3).

**Table 3:** Annual per-patient healthcare costs for a real-world type 2 diabetes mellitus population in the US in 2015, stratified by ASCVD status

	Patients with T2DM		
Cost category	Non-ASCVD *N* = 659,498 (54.8%)	ASCVD *N* = 543,098 (45.2%)	% Change in Cost (ASCVD vs non-ASCVD)
All patients with healthcare cost data, n (%)	562,185 (85.2)	474,271 (87.3)	–
18 to 44 y	80,315 (12.2)	14,278 (2.6)	–
45 to 64 y	385,936 (58.5)	216,423 (39.8)	–
≥ 65 y	95,934 (14.5)	234,570 (43.2)	–
Total healthcare cost, $, mean (SD)	9735 (24,213)	22,977 (53,672)	+ 136%
18 to 44 y	8294 (26,522)	20,135 (50,246)	+ 143%
45 to 64 y	9658 (22,723)	21,912 (50,954)	+ 127%
≥ 65 y	11,248 (27,668)	24,089 (56,138)	+ 114%
Total medical cost, $, mean (SD)	6079 (20,689)	17,849 (51,302)	+ 194%
18 to 44 y	5457 (18,647)	15,727 (45,441)	+ 188%
45 to 64 y	5786 (19,730)	16,143 (45,857)	+ 179%
≥ 65 y	7777 (25,452)	19,488 (54,434)	+ 151%
Total pharmacy cost, $, mean (SD)	3656 (10,467)	5128 (11,803)	+ 40%
18 to 44 y	2837 (15,169)	4408 (17,639)	+ 55%
45 to 64 y	3872 (9623)	5769 (13,207)	+ 49%
≥ 65 y	3471 (8720)	4600 (9867)	+ 33%
Outpatient office visit cost, $, mean (SD)	4227 (14,624)	10,349 (35,356)	+ 145%
18 to 44 y	3478 (12,816)	8390 (25,507)	+ 141%
45 to 64 y	4090 (13,548)	9314 (31,470)	+ 128%
≥ 65 y	5404 (19,377)	11,384 (38,907)	+ 111%
Inpatient hospital visit cost, $, mean (SD)	1312 (11,178)	5646 (27,483)	+ 330%
18 to 44 y	1187 (9471)	4961 (29,554)	+ 318%
45 to 64 y	1212 (11,149)	5115 (27,350)	+ 322%
≥ 65 y	1821 (12,519)	6159 (27,464)	+ 238%
Emergency room outpatient^a^ cost, $, mean (SD)	382 (1737)	925 (3660)	+ 142%
18 to 44 y	609 (2326)	1543 (4747)	+ 153%
45 to 64 y	343 (1529)	896 (3416)	+ 161%
≥ 65 y	347 (1925)	915 (3792)	+ 164%
Emergency room inpatient^b^ cost, $, mean (SD)	158 (3112)	928 (9087)	+ 487%
18 to 44 y	184 (3057)	834 (6211)	+ 353%
45 to 64 y	141 (3028)	818 (8310)	+ 480%
≥ 65 y	205 (3470)	1030 (9856)	+ 402%

*T2DM* Type 2 diabetes mellitus

Currency reflects 2015 US$

*ASCVD* Atherosclerotic cardiovascular disease, *SD* Standard deviation, *y* Years

^a^Not resulting in inpatient admission

^b^Resulting in inpatient admission

**Fig. 3:**
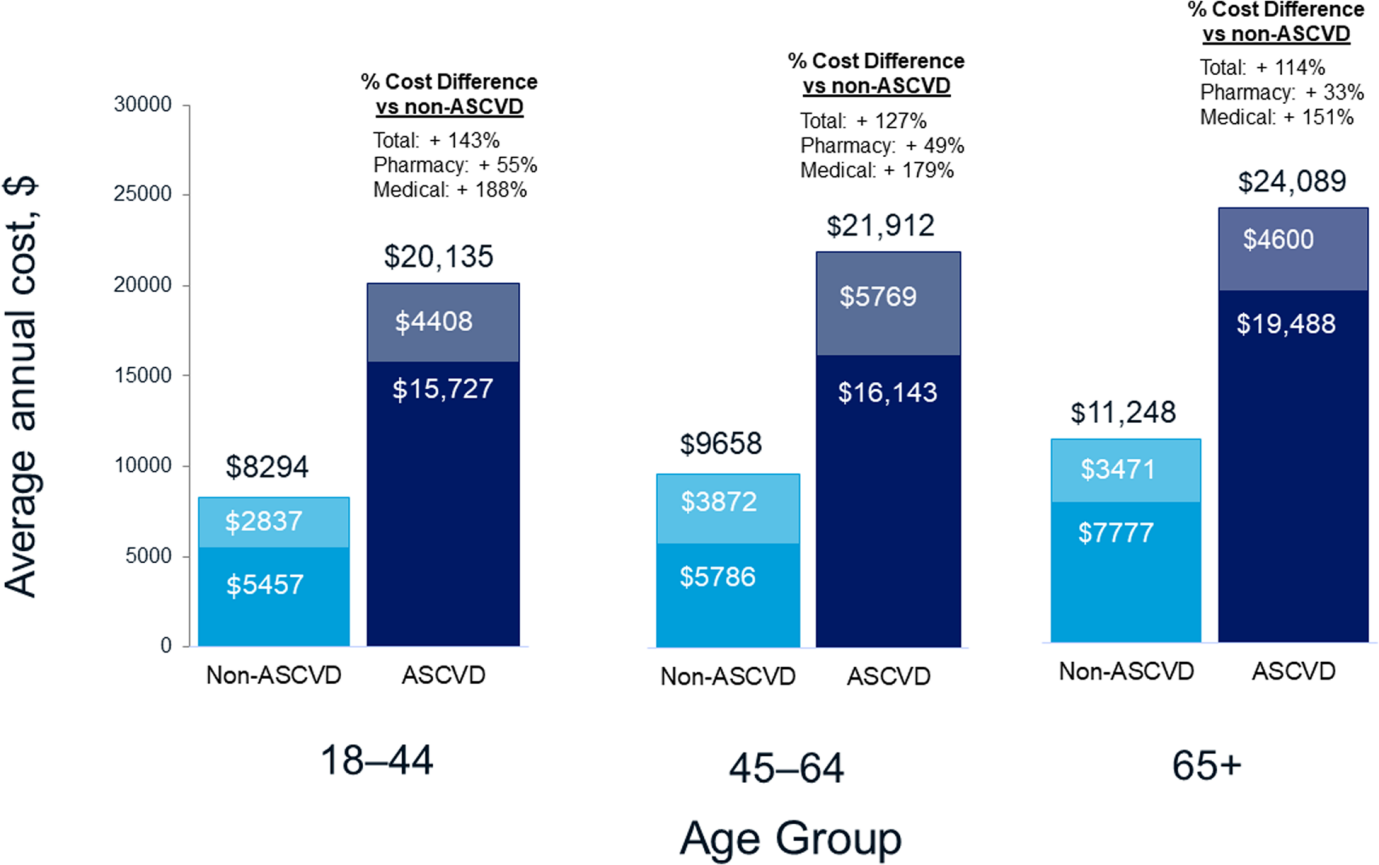
Average annual (2015) total healthcare costs per-patient with T2DM by age group and ASCVD status. Footnotes: *ASCVD* Atherosclerotic cardiovascular disease; *T2DM* Type 2 diabetes mellitus. Bars = total costs; Darker shading = medical costs; Lighter shading = outpatient pharmacy costs

For the entire study population with cost data, regardless of ASCVD status, mean annual per patient total healthcare costs during 2015 increased with increasing age group, from $10,082 in the youngest group (18–44 years) to $14,061 in the middle group (45–64 years) and $20,460 in the oldest group (≥ 65 years). Within each age category, mean annual costs were markedly higher in patients with ASCVD as compared to those without ASCVD (Table 3**,** Fig. 3). The percent difference in mean annual costs for patients with ASCVD versus those without ASCVD within cost categories ranged from + 33% to + 487% (Table 3). The absolute difference in mean annual per patient total healthcare costs for patients with ASCVD versus patients without ASCVD was $11,841 in the 18–44 year age group ($20,135 vs $8294), $12,254 in the 45–64 year age group ($21,912 vs $9658), and $12,841 in the ≥ 65 year age group ($24,089 vs $11,248). For the major cost categories (total healthcare costs, total medical costs, total pharmacy costs, outpatient office visits), the percentage differences for ASCVD vs non-ASCVD cohorts were largest for the youngest age categories (18–44 years) as compared with the other two older age categories. Compared to non-ASCVD patients, total annual healthcare costs for patients with ASCVD were 143%, 127%, and 114% higher in the 18–44, 45–64, and ≥ 65 year old age groups, respectively; medical costs were 188%, 179%, and 151% higher, and pharmacy costs were 55%, 49%, and 33% higher, respectively (Fig. 3).

Similar results for total healthcare costs were observed using a linear regression model performed on data from a cohort of 757,996 patients propensity-score matched 1:1 (ASCVD:non-ASCVD) on age, gender, region, and insurance type (*n* = 378,998) in each group; characteristics presented in Additional file 2: Table S2). Mean annual total per patient healthcare costs in these matched cohorts remained markedly higher in the ASCVD group ($22,480) as compared to the non-ASCVD group ($10,243). Similar findings were noted for each individual cost category (mean annual costs): total medical costs ($17,021 vs $6479, including inpatient [$5369 vs $1438], ER inpatient [$856 vs $168], ER outpatient [$919 vs $343], and outpatient visits [$9877 vs $4531]) and total pharmacy costs ($5459 vs $3764) (Additional file 3: Table S3).

## Discussion

With a dataset encompassing 1.2 million patients with T2DM, this is one of the largest analyses to have assessed total healthcare utilization and costs in patients with T2DM in the US stratified by the presence or absence of ASCVD. It found that almost one-half (45.2%) of all adults with T2DM had concomitant ASCVD during the analysis period of 2014–2015. As would be expected, the prevalence of ASCVD increased with age, from 15% of the youngest cohort (18–44 years) to 71% of the oldest cohort (≥ 65 years). Patients with ASCVD used more healthcare resources and had higher costs related to healthcare for every evaluated resource category (outpatient office visits, inpatient hospital admissions, ER visits) than patients without ASCVD. The mean total healthcare cost per patient with T2DM with concomitant ASCVD ($22,977) was more than double the mean total healthcare cost per patient with T2DM but without ASCVD ($9735), with concomitant ASCVD adding a mean $13,242 per patient per year. Medical (non-pharmacy) costs were almost tripled in patients having T2DM with ASCVD, while pharmacy costs were about 40% higher.

These findings corroborate those from a smaller (*N* = 138,018) US study by Mehta et al. which used linked EMR-claims data and identified CVD (defined as a history of stroke, transient ischemic attack, MI, unstable angina, or coronary revascularization) in 12% of patients with T2DM. This was lower than the 45.2% prevalence of ASCVD noted in the present study, for which the definition of ASCVD included additional indications of peripheral arterial disease or acute coronary syndrome, which were the ASCVD indications with highest prevalence. Mehta et al. reported that unadjusted monthly total treatment costs per patient were significantly higher, by almost twice, for patients with CVD compared with patients without CVD ($2655 vs $1435) [13], similar to the 2.4-times higher costs for patients with versus without ASCVD in our study. Interestingly, both the Mehta study and our study observed an apparent disproportionate increase in ASCVD-related cost burden in younger relative to older age groups. In our study, the youngest age group (18–44 years) demonstrated the largest difference in costs between patients with vs without concomitant ASCVD (+ 143%); corresponding costs differences in the 45–64 and ≥ 65 year age groups were + 127% and 114%, respectively. Although not directly comparable with unadjusted costs in the present study, it is worth noting that adjusted costs associated with CVD in the Mehta 2018 study were the highest (56% higher) in patients aged less than 45 years and lowest (2% higher) in patients aged more than 64 years [13].

A study by Johnston et al. [11] used MarketScan data (2009–2010) to quantify the cost of major adverse cardiovascular events (MACE; MI and stroke) in 1,415,598 patients with T2DM. Patients were assigned risk categories (lowest, medium, highest) based upon age, sex, number of baseline claims for atherosclerosis, stroke, MI, unstable angina, coronary revascularization, heart failure, dyslipidemia, hypertension, and tobacco use disorder, and were followed 301 to 343 days for occurrence of MACE. Healthcare costs were substantially higher for 10,399 (0.73%) patients with MACE, although costs varied with CVD risk category and payer (commercial, Medicare with supplemental insurance, Medicaid). Among patients with the highest CVD risk, expected per patient per month costs associated with MACE events alone (not all-cause healthcare) ranged from $9574–$18,727, depending on insurance type.

Li et al. [12] analyzed data from 7109 patients with T2DM from the Translating Research Into Action for Diabetes study and used a generalized linear regression model to estimate associations between medical costs and various patient characteristics, including comorbidities. Based on modeling findings, the presence of coronary heart disease increased direct medical costs 1.8-fold in patients with T2DM.

Thus, existing data confirm the substantial cost burden of ASCVD and related comorbidities in patients with T2DM, and there is growing momentum to address cardiovascular complications within the scope of diabetes care. The primary goals of T2DM management, in addition to maintenance of patient quality of life, are to prevent the development or progression of diabetes-related complications through glycemic control and management of cardiovascular risk [21]; as such, the treatment of patients with diabetes is evolving in order to focus more intently on CVD risk reduction. While some older pharmacological agents used to manage diabetes, such as sulfonylureas, may increase CV risk [22, 23], newer classes of agents such as GLP-1RAs and SGLT2is have been shown to reduce the risk of MACE in patients with established CVD independent of their glucose-lowering benefits [24–27]. Thus, current ADA guidelines recommend that, in patients with T2DM and established CVD who require therapy beyond lifestyle management and metformin, agents with proven cardiovascular benefit should be incorporated as part of glycemic management. Antidiabetic agents with dual impact on both glycemia and CVD risk reduction have the potential to improve health outcomes, limit the number of drugs administered, and possibly reduce overall costs.

Of note, 15% of patients in the youngest age category in the current study (18–44 years) had established ASCVD, and 36% of those in the middle age group (45–64 years). The development of serious comorbidities at a relatively young age can translate into substantial medical cost implications over time and aggressive preventive measures are warranted for both economic and health reasons. Some data suggest that early-onset T2DM in younger patients carries a particularly high cardiovascular risk, including a 14-fold increased risk of MI compared with matched, non-T2DM controls [27].

The findings of this analysis, while robust, are not without limitations. Despite the large nationwide sample, no data were obtained from patients insured by Medicaid or from uninsured patients, thus the results may not be completely generalizable to the entire US population. Nonetheless, generalizability to the US population is enhanced by the sheer size of the study sample (over one-half million patients in each cohort) and diverse geographical sampling distribution. Substantial differences in insurance coverage patterns on healthcare resource use and costs were not assessed. As the prevalence of ASCVD increases with age group and as commercial insurance is replaced by Medicare in older patients, it is possible that the availability of healthcare resources and pharmacological therapies would not be equivalent between insurance carriers across age cohorts. There was a substantial difference between groups in the distribution of types of insurance carriers; approximately 50% of patients with concomitant ASCVD had commercial insurance, as compared to 84% of patients without ASCVD. Not all patients had detailed cost data available; yet this situation was similar for both cohorts and reflected < 15% of either group, and thus should not have impacted the study findings substantially. Propensity score matching was performed based on patient age, sex, region, and insurance, and did not factor in comorbidity variables. It should be noted that the propensity score-matched ASCVD cohort had higher mean DCSI (2.41 vs 0.85) and CCI (2.93 vs 1.76) scores as compared to the non-ASCVD cohort, suggesting greater overall comorbidity burden in the ASCVD cohort. The excess mean prevalence of any non-ASCVD comorbidity/complication in the matched ASCVD cohort versus the non-ASCVD cohort was less than 10% with the exception of hypertension: hypertension (10.1%), dyslipidemia (6.8%), metabolic (2.2%), nephropathy (7.3%), and retinopathy (2.6%). While these differences appear to be relatively minor, a confounding effect of higher comorbidity prevalence in the ASCVD cohort on cost differences between the groups cannot be ruled out and the reader should interpret the study findings with this in mind. Finally, the current analysis is dependent upon the coding practices of providers, which may be subject to coding error, and upon ICD codes alone to document ASCVD and comorbidities, which could also have been subject to coding error. Indirect costs, such as loss of productivity, were not captured.

## Conclusions

These findings indicate significantly higher utilization of healthcare resources and associated costs in patients having T2DM with ASCVD compared to patients having T2DM without ASCVD. The high prevalence and excessive healthcare costs of concomitant T2DM and ASCVD provide compelling incentives to find more cost-efficient strategies to effectively manage patients with these concomitant diagnoses. Further research is required to evaluate the potential economic implications of newer antidiabetic therapies with proven CVD benefits in patients with T2DM.

## Supplementary information

**Additional file 1: Table S1.** Diagnoses factored into the Diabetes Complications Severity Index (DCSI) score [18].

**Additional file 2: Table S2.** Characteristics of propensity score-matched cohorts^a^.

**Additional file 3: Table S3.** Annual per-patient healthcare costs for propensity score-matched cohorts^a^ with type 2 diabetes mellitus population in the US in 2015, stratified by ASCVD status.

## Data Availability

The data that support the findings of this study are available from IBM/Truven. Restrictions apply to the availability of these data, which were used under license for this study. Data are available Wayne Weng with the permission of IBM/Truven.

## Abbreviations

ASCVD: Atherosclerotic cardiovascular disease
CCI: Charlson Comorbidity Index
CVD: Cardiovascular disease
DCSI: Diabetes Complications Severity Index
EMR: Electronic medical records
ER: Emergency room
MACE: Major adverse cardiovascular events
MI: Myocardial infarction
SD: Standard deviation
T2DM: Type 2 diabetes mellitus
