# Supplementary material for: Impact of atherosclerotic cardiovascular disease on healthcare resource utilization and costs in patients with type 2 diabetes mellitus in a real-world setting
Source: Clin Diabetes Endocrinol. 2020 Mar 4;6:5. doi: 10.1186/s40842-019-0090-y (PMC7057457; doi:10.1186/s40842-019-0090-y)
Supplement: Supplementary file 1 — Additional file 1: Table S1. Diagnoses factored into the Diabetes Complications Severity Index (DCSI) score [18]. [file 40842_2019_90_MOESM1_ESM.docx]

**Supplemental Table 1.** Diagnoses factored into the Diabetes Complications Severity Index (DCSI) score [18].

| **Complication Category** | **Specific Diagnoses Included** | **DCSI Value** |
| --- | --- | --- |
| **Retinopathy** | Diabetic ophthalmologic disease | 1 |
|  | Background retinopathy | 1 |
|  | Other retinopathy | 1 |
|  | Retinal edema | 1 |
|  | CSME | 1 |
|  | Other retinal disorders | 1 |
|  | Proliferative retinopathy | 2 |
|  | Retinal detachment | 2 |
|  | Blindness | 2 |
|  | Vitreous hemorrhage | 2 |
| **Nephropathy** | Diabetic nephropathy | 1 |
|  | Acute glomerulonephritis | 1 |
|  | Nephrotic syndrome | 1 |
|  | Hypertension, nephrosis | 1 |
|  | Chronic glomerulonephritis | 1 |
|  | Nephritis/nephropathy | 1 |
|  | Chronic renal failure | 2 |
|  | Renal failure NOS | 2 |
|  | Renal insufficiency | 2 |
|  | Urine protein ≥30 mg/g of creatinine, *or* (+) dipstick protein *or* serum creatinine ≥1.5 mg/dL | 1 |
|  | Serum creatinine >2.0 mg/dL | 2 |
| **Neuropathy** | Diabetic neuropathy | 1 |
|  | Amyotrophy | 1 |
|  | Cranial nerve palsy | 1 |
|  | Mononeuropathy | 1 |
|  | Charcot’s arthropathy | 1 |
|  | Polyneuropathy | 1 |
|  | Neurogenic bladder | 1 |
|  | Autonomic neuropathy | 1 |
|  | Gastroparesis/diarrhea | 1 |
|  | Orthostatic hypotension | 1 |
| **Cerebrovascular** | TIA | 1 |
|  | Stroke | 2 |
| **Cardiovascular** | Atherosclerosis | 1 |
|  | Other IHD | 1 |
|  | Angina pectoris | 1 |
|  | Other chronic IHD | 1 |
|  | Myocardial infarction | 2 |
|  | Ventricular fibrillation, arrest | 2 |
|  | Atrial fibrillation, arrest | 2 |
|  | Other ASCVD | 1 |
|  | Old myocardial infarction | 2 |
|  | Heart failure | 2 |
|  | Atherosclerosis, severe | 2 |
|  | Aortic aneurysm/dissection | 2 |
| **Peripheral vascular disease** | Diabetic PVD | 1 |
|  | Other aneurysm, LE | 1 |
|  | PVD | 1 |
|  | Foot wound + complication | 1 |
|  | Claudication, intermittent | 1 |
|  | Embolism/thrombosis (LE) | 2 |
|  | Gangrene | 2 |
|  | Gas gangrene | 2 |
|  | Ulcer of lower limbs | 2 |
| **Metabolic** | Ketoacidosis | 2 |
|  | Hyperosmolar | 2 |
|  | Other coma | 2 |

A score is assigned to each of the seven complication categories based on the highest score present for any of the specific diagnoses in that category. Possible scores per category are 0, 1, or 2 (except neuropathy, which can only be 0 or 1). The total DCSI score is a sum of the seven category scores (maximum score = 13).
