# Supplementary material for: Impact of atherosclerotic cardiovascular disease on healthcare resource utilization and costs in patients with type 2 diabetes mellitus in a real-world setting
Source: Clin Diabetes Endocrinol. 2020 Mar 4;6:5. doi: 10.1186/s40842-019-0090-y (PMC7057457; doi:10.1186/s40842-019-0090-y)
Supplement: Supplementary file 2 — Additional file 2: Table S2. Characteristics of propensity score-matched cohortsa. [file 40842_2019_90_MOESM2_ESM.docx]

**Supplemental Table 2**. Characteristics of propensity score-matched cohorts^a^

|  | Patients with type 2 diabetes mellitus | |
| --- | --- | --- |
| Variable | Non-ASCVD  N=378,998 | ASCVD  N=378,998 |
| Age, y, mean (SD) | 61.2 (10.1) | 61.3 (10.0) |
| Age category, n (%) |  |  |
| 18–44 y | 17,029 (4.5) | 17, 029 (4.5) |
| 45–64 y | 250,179 (66.0) | 250,179 (66.0) |
| ≥65 y | 111,790 (29.5) | 111,790 (29.5) |
| Sex, n (%) |  |  |
| Female | 188,095 (49.6) | 184,479 (48.7) |
| Male | 190,903 (50.4) | 194,519 (51.3) |
| Region of US, n (%) |  |  |
| North Central | 93,929 (24.8) | 94,638 (25.0) |
| Northeast | 79,000 (20.8) | 83,305 (22.0) |
| South | 165,095 (43.6) | 162,495 (42.9) |
| West | 40,129 (10.6) | 37,639 (9.9) |
| Unknown | 845 (0.2) | 921 (0.2) |
| Insurance, n (%) |  |  |
| Commercial | 273,552 (72.2) | 273,526 (72.2) |
| Medicare | 105,446 (27.8) | 105,472 (27.8) |
|  |  |  |
| ASCVD diagnosis^b^, n (%) |  |  |
| Acute coronary syndrome | — | 207,624 (54.8) |
| Angina | — | 73,537 (19.4) |
| Myocardial infarction | — | 54,801 (14.5) |
| Peripheral arterial disease | — | 189,946 (50.1) |
| Revascularization | — | 57,108 (15.1) |
| Stroke | — | 133,538 (35.2) |
| Transient ischemic attack | — | 44,792 (11.8) |
| Hypertension, n (%) | 288,990 (76.3) | 327,505 (86.4) |
| Dyslipidemia, n (%) | 289,965 (76.5) | 315,754 (83.3) |
| Diabetes-related complications^c^, n (%) |  |  |
| Cardiovascular^d^ | 17,540 (4.6) | 198,279 (52.3) |
| Cerebrovascular | 0 (0.0) | 71,321 (18.8) |
| Metabolic^e^ | 44,044 (11.6) | 52,349 (13.8) |
| Nephropathy | 41,791 (11.0) | 69,167 (18.3) |
| Peripheral vascular^f^ | 9683 (2.6) | 65,149 (17.2) |
| Retinopathy | 41,900 (11.1) | 52,078 (13.7) |
| DCSI score, mean (SD) | 0.85 (1.25) | 2.41 (2.13) |
| CCI score, mean (SD) | 1.76 (1.46) | 2.93 (2.24) |

ASCVD, atherosclerotic cardiovascular disease; CCI, Charlson Comorbidity Index; DCSI, Diabetes Complications Severity Index; SD, standard deviation

Note: Age, sex, region, and insurance determined as of January 1, 2015. Comorbidities were captured by any appearance during 2014-2015.

^a^Cohorts matched by age, sex, region and insurance

^b^Defined by ADA 2017 guidelines. Patients could have more than one diagnosis.

^c^Comorbidities included in the Diabetes Complications Severity Index [18]

^d^As defined by the Diabetes Complications Severity Index [18], category includes diagnoses of atherosclerosis, other ischemic heart disease, angina, other chronic ischemic heart disease, myocardial infarction, ventricular fibrillation, arrest; atrial fibrillation, arrest; other ASCVD, old myocardial infarction, heart failure, atherosclerosis, severe; aortic aneurysm/dissection.

^e^Category includes ketoacidosis, hyperosmolar, and “other coma”

^f^Category includes any peripheral vascular disease, not limited to “peripheral arterial disease presumed to be of atherosclerotic origin” which was part of the “ASCVD” definition
