# Supplementary material for: Impact of atherosclerotic cardiovascular disease on healthcare resource utilization and costs in patients with type 2 diabetes mellitus in a real-world setting
Source: Clin Diabetes Endocrinol. 2020 Mar 4;6:5. doi: 10.1186/s40842-019-0090-y (PMC7057457; doi:10.1186/s40842-019-0090-y)
Supplement: Supplementary file 3 — Additional file 3: Table S3. Annual per-patient healthcare costs for propensity score-matched cohortsa with type 2 diabetes mellitus population in the US in 2015, stratified by ASCVD status. [file 40842_2019_90_MOESM3_ESM.docx]

**Supplemental Table 3.** Annual per-patient healthcare costs for propensity score-matched cohorts^a^ with type 2 diabetes mellitus population in the US in 2015, stratified by ASCVD status

|  | Propensity Score-matched Patients | | | Entire study Population, % Change in Cost (ASCVD vs non-ASCVD) |
| --- | --- | --- | --- | --- |
|  | Non-ASCVD  N=378,998 | ASCVD  N=378,998 | % Change in Cost (ASCVD vs non-ASCVD) |  |
| Patients with cost data, n (%) | 324,462 (85.6) | 325,051 (83.8) | --- | --- |
| Cost Category |  |  |  |  |
| Total healthcare cost, $, mean (SD) | 10,243 (24,489) | 22,480 (52,758) | +119% | +136% |
| Total medical cost, $, mean (SD) | 6479 (21,808) | 17,021 (49,963) | +163% | +194% |
| Total pharmacy cost, $, mean (SD) | 3764 (9261) | 5459 (12,723) | +45% | +40% |
| Outpatient office visit, $, mean (SD) | 4531 (15,887) | 9877 (33,532) | +118% | +145% |
| Inpatient hospital visit, $, mean (SD) | 1438 (11,134) | 5369 (27,590) | +273% | +330% |
| ER outpatient^b^, $, mean (SD) | 343 (1645) | 919 (3625) | +168% | +142% |
| ER inpatient^c^, $, mean (SD) | 168 (3476) | 856 (9012) | +410% | +487% |

ASCVD, atherosclerotic cardiovascular disease; ER, emergency room; SD, standard deviation

Currency reflects 2015 US dollars

^a^Cohorts matched by age, sex, region and insurance

^b^Not resulting in inpatient admission

^c^Resulting in inpatient admission
